# Analysis of strength property and pore characteristics of Taihang limestone using X-ray computed tomography at high temperatures

**DOI:** 10.1038/s41598-021-92928-z

**Published:** 2021-06-29

**Authors:** Shi Liu, Zhe Huang

**Affiliations:** 1 Aviation Engineering School, Air Force Engineering University, Xi’an, 710038 Shaanxi People’s Republic of China; 2 State Key Laboratory of Coal Resources and Safe Mining, China University of Mining and Technology, Xuzhou, 221116 Jiangsu People’s Republic of China

**Keywords:** Characterization and analytical techniques, Design, synthesis and processing, Microscopy

## Abstract

Rising temperature will cause the changes of pore characteristics and strength property in rock. This research takes the limestone produced in Taihang Mountains as the research object, and performs high-temperature treatment within 25–1000 °C. The high-resolution X-ray computed tomography (CT) scanning test method is used to visually reconstruct the three-dimensional image of the sample, and obtain the spatial distribution status of the mesoscopic parameters of the bones, pores/cracks, etc. The results show that when the temperature exceeded 700 °C, the samples appeared milky white in appearance and as the temperature increased, the color gradually turned white, macroscopic cracks began to appear on the surface, while the meso-pores connected rapidly, reflecting a typical progressive destruction process from inside to outside. The change law of volume porosity with temperature has a consistent trend with that of the apparent morphology of the sample. Similarly, the mechanical test results suggest that 700 °C is also the turning temperature for strength deterioration and brittle-plastic transformation of sample. Based on the results of high-temperature test, CT test and mechanical test, there are enough evidences to show that, for the limestone sample, 700 °C is probably to be the mutation temperature of physical–mechanical behavior.

## Introduction

As a typical extreme geological environment for deep underground rocks, the influence of high temperature on rock strength, deformation, and stability has become a popular topic in the field of rock mechanics and engineering^1–5^. Complex physical and chemical changes occur inside rock under high-temperature action^2–4,6–9^, the strength indexes of different types of rocks show different temperature effects, and even for the same type of rock, in different geological conditions, its temperature effect will vary significantly^10–13^. Ground temperature is an important factor affecting the strength properties of rocks that cannot be ignored. The current ground temperature for various types of drilling can reach hundreds of degrees Celsius. For underground rock engineering, the storage of oil and gas requires in-depth analysis of the influence of the temperature field on rock strength. For example, during the underground storage of radioactive nuclear waste, the temperature of the surrounding rock can reach 300 °C due to decay. When coal is gasified underground, the surrounding rock temperature is as high as 1000 °C. Therefore, the comprehensive study of the high-temperature strength of rocks has become an important research direction in the fields of rock mechanics and engineering, which will provide scientific theoretical basis and method support for the design, construction, and stability analysis of underground rock engineering under high-temperature environment. Rock can be regarded as a multi-phase composite structure containing many internal random defects^14–17^. The effect of temperature will cause the development, growth, and extension of various forms of microdefects dispersed in the material. Such microdefects have different manifestations at different levels of the evolution process^18–22^: (1) In geotechnical engineering, they manifest as various natural disasters, such as slope instability, landslides, and rock bursts; (2) In the laboratory, they appear as the loading deformation and failure of the rock sample. However, due to the extreme complexity of the pre-existing geological environment, the spatial distribution of rock skeletons, pore structures, etc., are extremely complicated. How to accurately represent the true shape of the rock spatial structure has always troubled researchers in related fields. At present, X-ray computed tomography method can achieve nondestructive observation of any cross section of the material^23–28^, the obtained perspective projection data after reconstruction can directly represent the three-dimensional information of the internal microstructure of the object^29–31^, and this technology has been increasingly used in the mesoscopic observation of geotechnical materials^32–37^.

To explore the changing trend of microstructure characteristics and the strength behavior of rock with temperature, this research takes the limestone collected from China’s Taihang Mountains as the research object, and carries out three types of tests: high-temperature test, CT test and mechanical test. The high-temperature treatment range is controlled at 25–1000 °C (25, 100, 200, 300, 400, 500, 600, 700, 800, 900, and 1000 °C). It is found that high temperature has a significant influence on the rock macroscopic morphology. An X-ray three-dimensional microscope is used to scan the limestone samples after different high-temperature treatments, and reconstruct the three-dimensional images. Using image display and quantitative analysis of reconstructed data, the quantitative description of the pores/cracks is realized, and the functional relationship between temperature and porosity is established. Based on the uniaxial mechanical test results, the strength characteristics and the change laws of brittle-plastic transformation of samples with temperature are explored.

## High-temperature test

### Sample preparation

**Figure 1:**
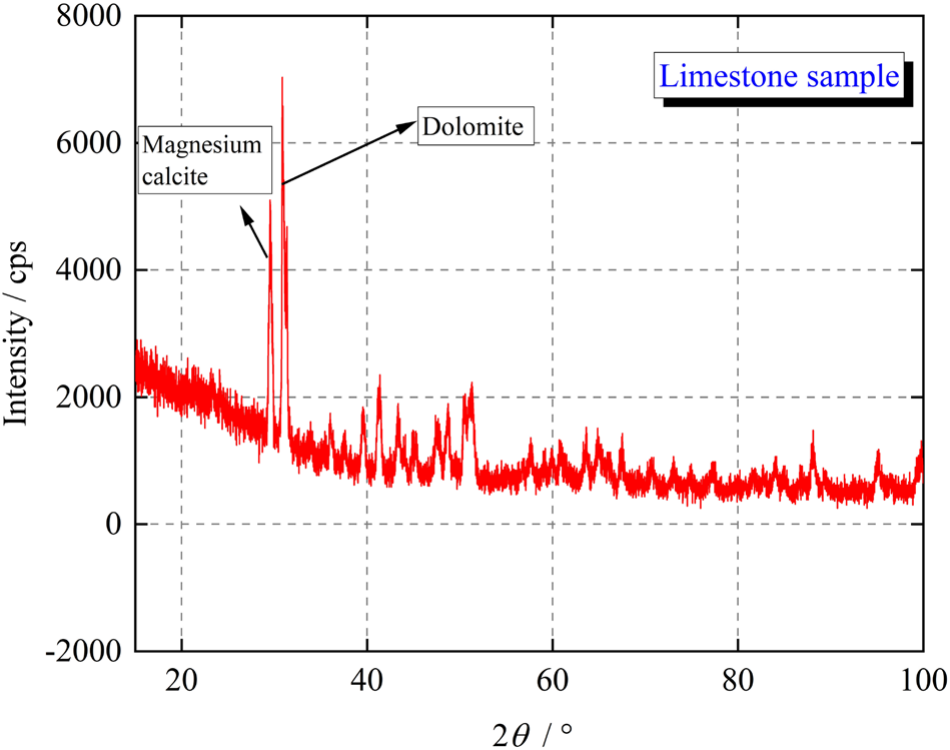
XRD test results of limestone samples.

The rock sample used in the experiment was limestone taken from the Northern Henan section of Taihang Mountains, a large mountain range in northern China, with an altitude between 1000 and 3500 m. The Taihang Mountains are an important geographical boundary that runs in a northeast to southwest direction and belongs to an uplift zone of the giant basin-ridge tectonic system in eastern China formed by the activities of the Cenozoic east Asian rift. The limestone minerals in the area are widely distributed and rich in resources, and the hydrological and engineering geological condition is simple. Limestone was formed in the shallow sea environment of the Cambrian period 500 million years ago. The XRD test results are shown in Fig. 1. It is mainly fine-grained and massive, with calcite and dolomite accounting for 97% to 98%, sand debris accounting for 0.1% to 1%, and quartz accounting for 0.5 to 1%. Among them, fine-crystalline calcite is mostly distributed in clumpy aggregates; dolomite is unevenly distributed in particulate aggregates. The apparent morphology of the limestone sample was blue-gray, with a density of 2.69 g/cm^3^. The sample size was a cube of 70 mm × 70 mm × 70 mm, the parallelism of the upper and lower surfaces of the specimen were within 0.05 mm, and the flatness of the surface was within 0.02 mm. To ensure the uniform nature of the tested rock samples, a non-metallic ultrasonic velocimeter was used to measure the ultrasonic longitudinal wave velocity of the samples, and those with obviously abnormal longitudinal wave velocity were excluded. The average longitudinal wave velocity of the selected limestone samples was 4200–4500 m/s.

### Influence of high temperature on samples

**Figure 2:**
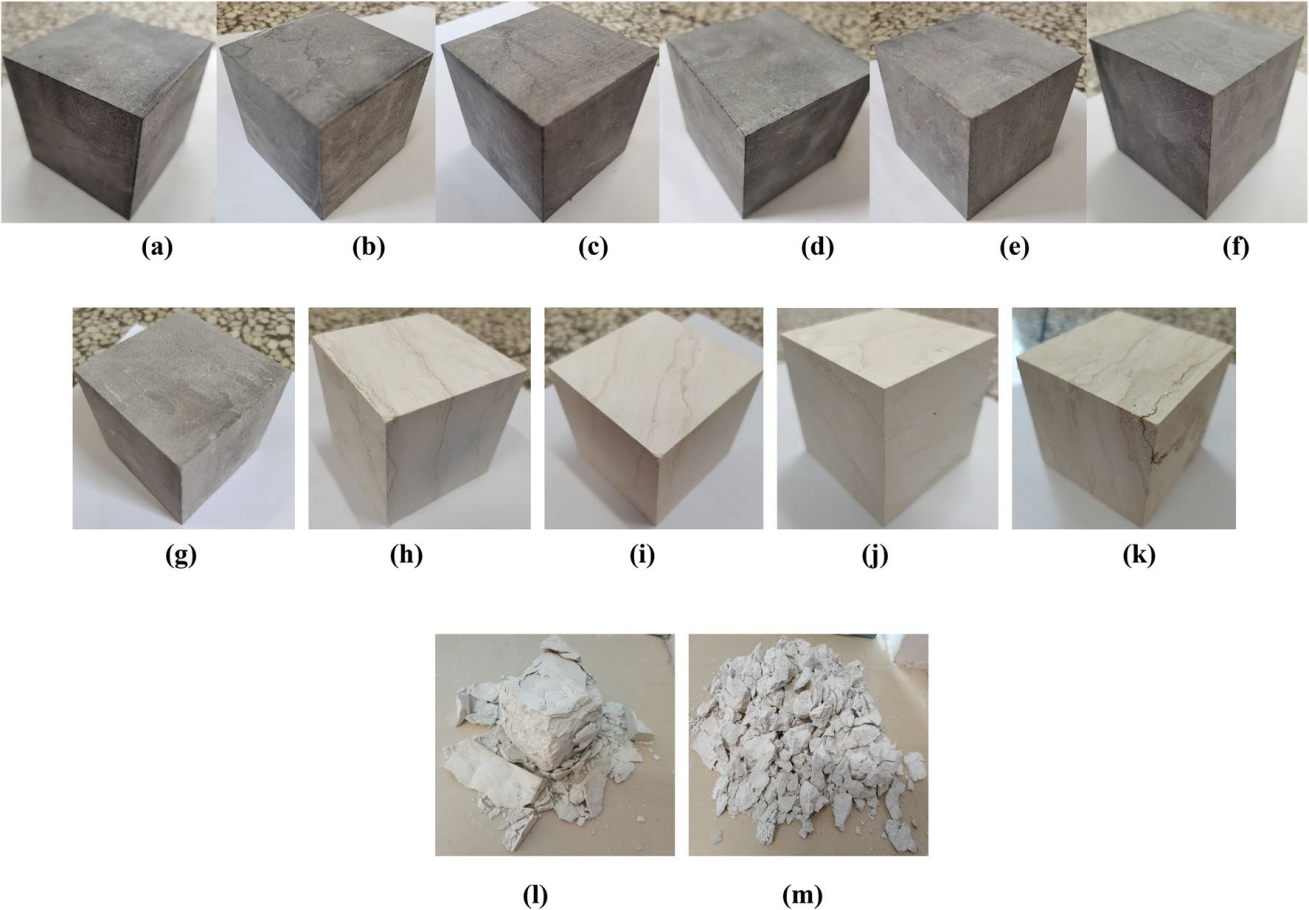
Apparent morphology of limestone samples after high-temperature treatment: (**a**) 25 °C; (**b**) 100 °C; (**c**) 200 °C; (**d**) 300 °C; (**e**) 400 °C; (**f**) 500 °C; (**g**) 600 °C; (**h**) 700 °C; (**i**) 800 °C; (**j**) 900 °C; (**k**) 1000 °C; (**l**) 1000 °C-72 h; (**m**) 1000 °C-120 h.

The limestone samples were heated in a high-temperature box, and the heating temperatures were set to 25, 100, 200, 300, 400, 500, 600, 700, 800, 900, and 1000 °C, a total of 11 groups. The samples were heated at a heating rate of 10 °C/min and kept constant for 2 h after reaching the target temperature to ensure that they were uniformly heated. Samples were then cooled to room temperature in a high-temperature box. Three samples were tested in each group. The apparent morphology of typical high-temperature limestone is shown in Fig. 2. The results of the previous high-temperature heating tests on marble and sandstone have shown that the physical properties of rock samples have a temperature threshold^12,13^, and the threshold of different kinds of rocks is different. For the limestone sample in this research, if judged qualitatively from the apparent morphology after high-temperature treatment, the sample experienced obvious naked eye changes at around 700 °C. When the heating temperature was in the range of 25–600 °C, the change in color appearance of the limestone sample did not appear obvious. When the temperature exceeded 700 °C, the sample appeared milky white, and, as the temperature increased, the surface began to crack, indicating that high temperature aggravated the damage to the rock sample.

Because high temperatures have an obvious destructive effect on limestone samples, it is difficult for samples to maintain long-term stability in the natural environment after high-temperature treatment. This is especially evident for limestone samples after treatment at 1000 °C. Figure 2-(l) and (m) show the apparent morphology of a limestone sample exposed to 1000 °C after standing for 72 h and 120 h at room temperature, respectively. Obviously, the sample could not maintain the basic cubic shape after 72 h; the outer surface fell off in a large area, and only one main core remained. After standing for 120 h, the sample completely collapsed, and the load-bearing capacity was completely lost in the absence of any external loading, and it degenerated into loose particles only in the natural environment. These results show that high temperature had a huge impact on the microstructural characteristics of the limestone sample, and these effects could not be reflected by the macro test.

**Figure 3:**
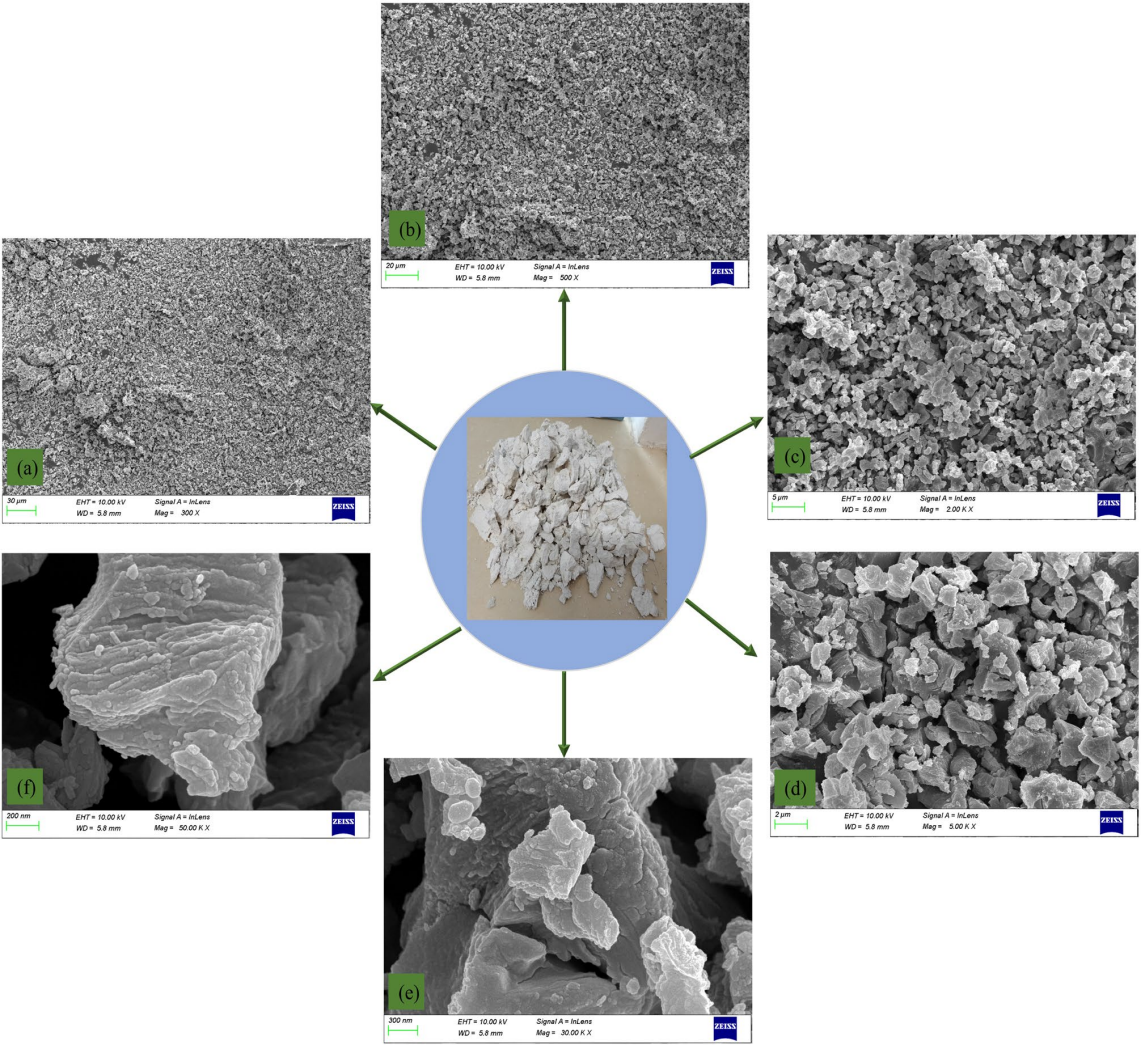
SEM morphology of marble samples exposed to 1000 °C after standing for 120 h at room temperature: (**a**) × 300; (**b**) × 500; (**c**) × 2000; (**d**) × 5000; (**e**) × 30,000; (**f**) × 50,000.

The SEM images of the limestone sample after experiencing a high temperature of 1000 °C and standing at room temperature for 120 h are shown in Fig. 3. Three typical SEM images with different magnifications (300, 500, 2000, 5000, 30,000, and 50,000 times) are selected. The images can qualitatively reflect the microstructure morphology of the sample after the ultra-high temperature treatment. It is obvious that the limestone sample has only experienced high-temperature treatment without external load, but the deterioration of the microstructure is very serious. There is almost no cohesive force between mineral particles, the particles are loosely packed, the pores/cracks between each other are large, and the fracture characteristics along the crystal are prominent. It is due to the significant changes in the cohesion between the crystals and the friction between the mineral particles that the sample does not have the load-bearing capacity in the macroscopic view. SEM images can only reflect the localized characteristics, which is representative but not universal. Only mesoscale nondestructive testing, especially visual reconstruction, can intuitively demonstrate the effect of high temperature. X-ray three-dimensional microscope scanning test methods were used to explore the effect of high temperature on rock samples in this work.

## X-ray three-dimensional microscope scanning test

### CT test equipment

**Figure 4:**
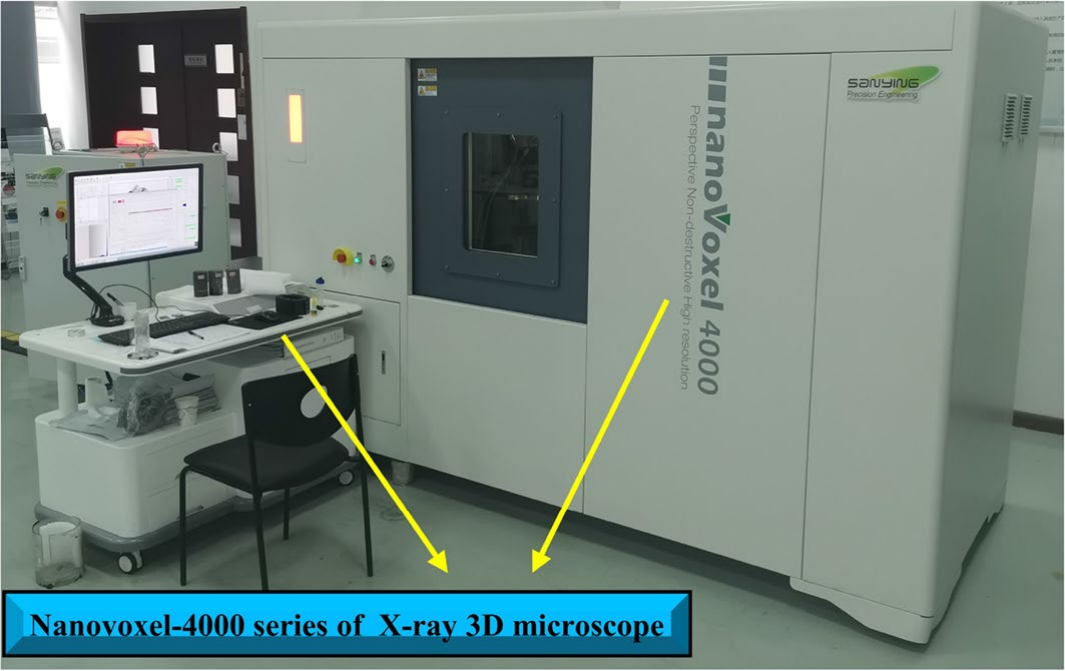
Nanovoxel-4000 series of high-resolution X-ray 3D microscope.

The instrument used in this test was the nanoVoxel-4000 series high-resolution X-ray three-dimensional microscope, produced by Sanying Precision Instruments Co., Ltd. The instrument is shown in Fig. 4. The instrument can perform high-quality image to obtain the true structure of the sample, providing a new high-resolution 3D nondestructive testing solution. The basic testing principle was as follows: A cone X-ray beam emitted by a microfocus ray source was used to penetrate the object and project it on the detector; the sample was rotated 360° relative to the ray source and detector to collect thousands of frames of X-ray attenuation images; then, 3D reconstruction was performed using the computer tomography reconstruction method to obtain a three-dimensional model of the sample. The CT image reflects the information about the energy attenuation of X-rays in the process of penetrating the object. On the macroscopic research scale, the relative density of the internal structure of the sample is positively correlated with the gray level of the CT image. The instrument was a high-resolution industrial CT, with a detailed resolution exceeding 2 μm, which could be combined with a high-resolution objective lens coupled detector and a large-field flat-panel detector to form a unique dual-optical X-ray imaging detection system. Considering the medium size of the limestone samples, the scanning accuracy could only be controlled at about 10^2^ μm, and one frame of image was collected every 0.25°. The specific test parameters are shown in Table 1.

**Table 1:** Basic test parameters.

Technical parameters	Resolution ratio/μm	Voltage/kV	Electric current/μA	Exposure time/s	Scanning time/h
Limestone sample	35.79	280	350	0.70	1

### Three-dimensional reconstruction visualization image of limestone sample

**Figure 5:**
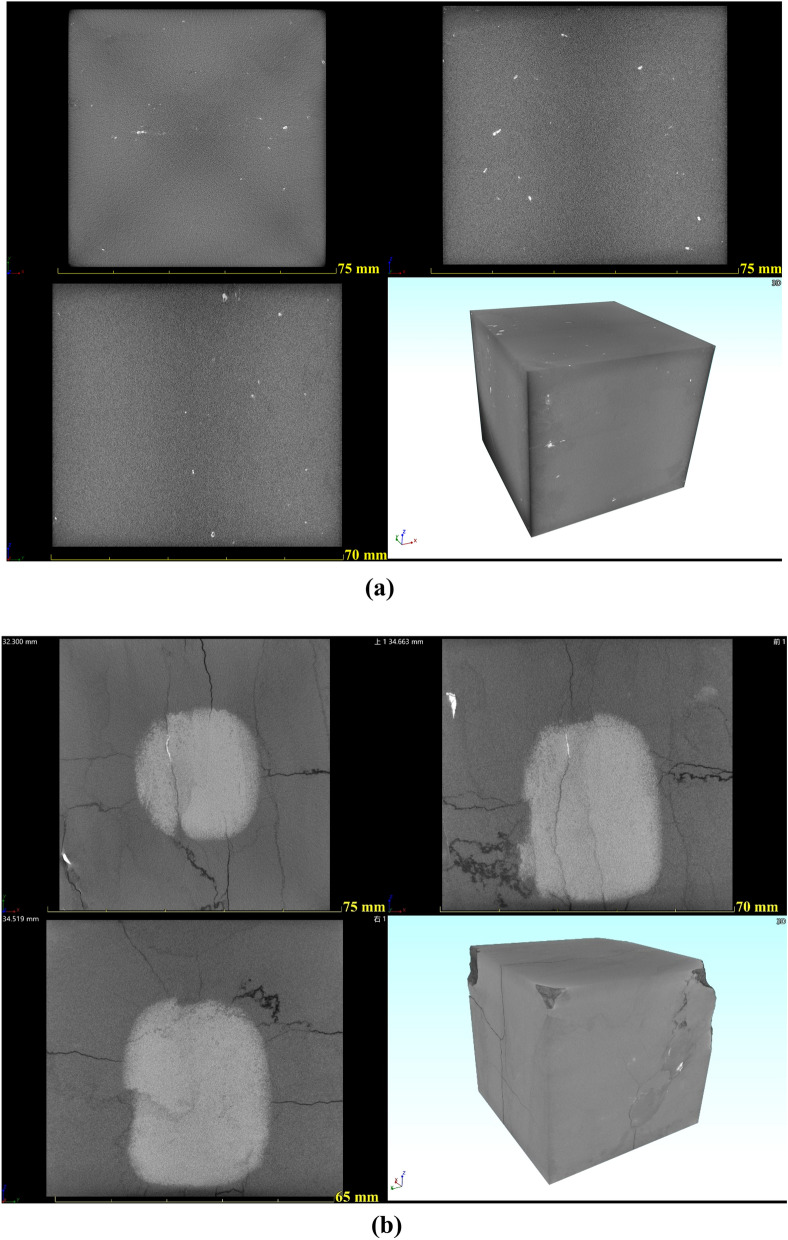
Four views of CT scan of limestone samples at typical temperatures: (**a**) 25 °C; (**b**) 1000 °C.

**Figure 6:**
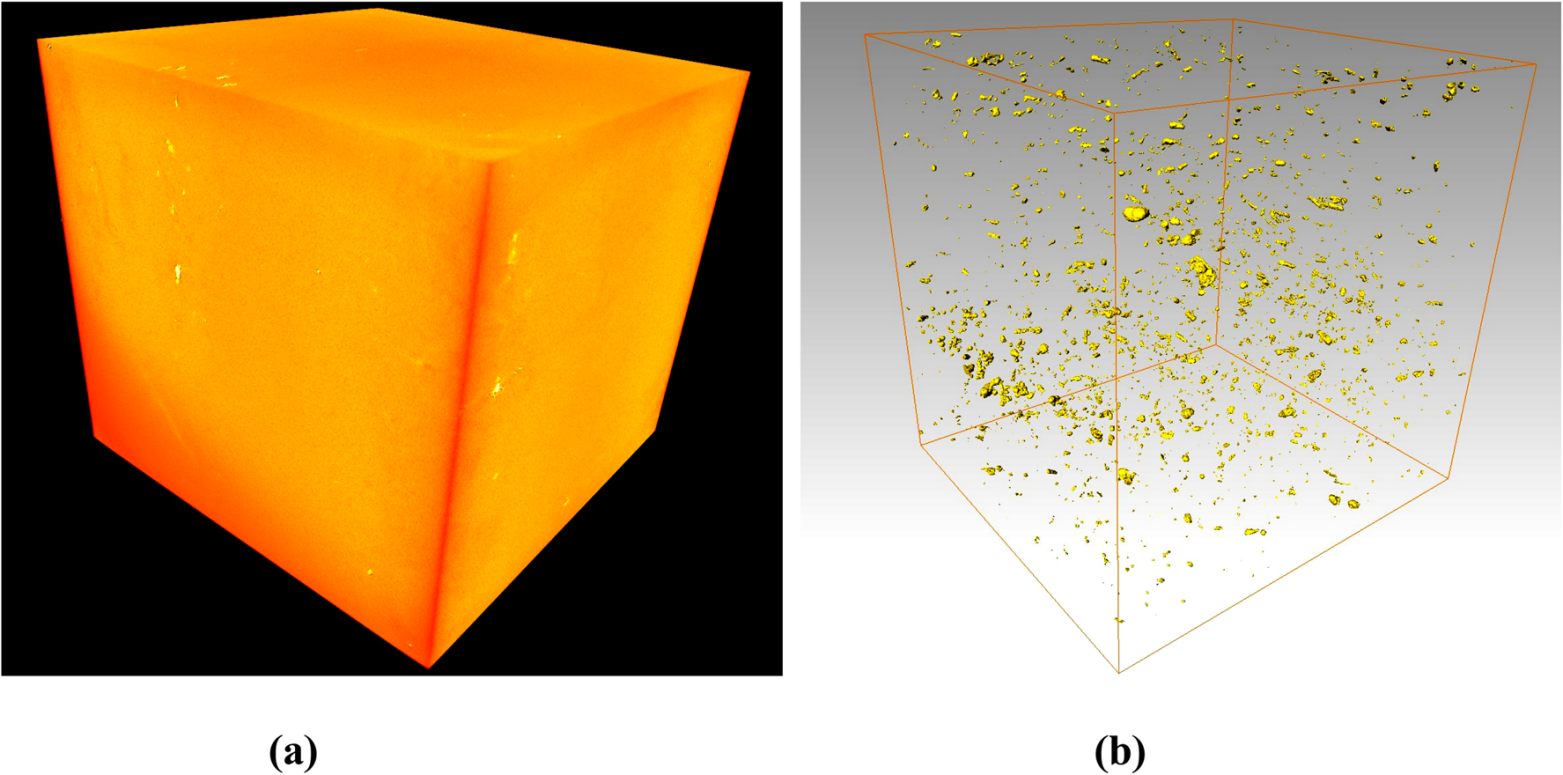
Three-dimensional visualization image of limestone sample under normal temperature: (**a**) Three-dimensional reconstruction rendering image; (**b**) Three-dimensional distribution map of high-density material.

The reconstruction software Voxel Studio Recon was used to carry out algorithm reconstruction, image correction, and the processing of the scanned data. Based on a series of digital image processing methods, such as image denoising, image enhancement, image segmentation, image recognition, etc., the three-dimensional reconstructed images of limestone samples after different high-temperature treatments were obtained. Figure 5 shows four views of the CT scan of the sample under normal temperature (25 °C) and after a high-temperature treatment of 1000 °C. Image display and quantitative analysis on the reconstructed data of the sample were performed to realize the CT feature recognition of the image and obtain the spatial distribution status of the rock sample bones, pores/cracks, and other meso-parameters. Figure 6 shows the 3D reconstruction visualization image of the limestone sample under normal temperature conditions. It is important to point out that, due to the large sample size and the test resolution of 35.70 μm, it is difficult to extract pores smaller than the resolution. According to the test results of limestone samples under normal temperature, the bright white color is a high-density substance. Quantitative statistics of high-density materials were obtained by threshold segmentation, and the percentage of the total sample volume was 0.09%. The overlap of high-density materials and pores was difficult to distinguish. Therefore, this research only lists the visual images of limestone samples processed from 700 to 1000 °C.

**Figure 7:**
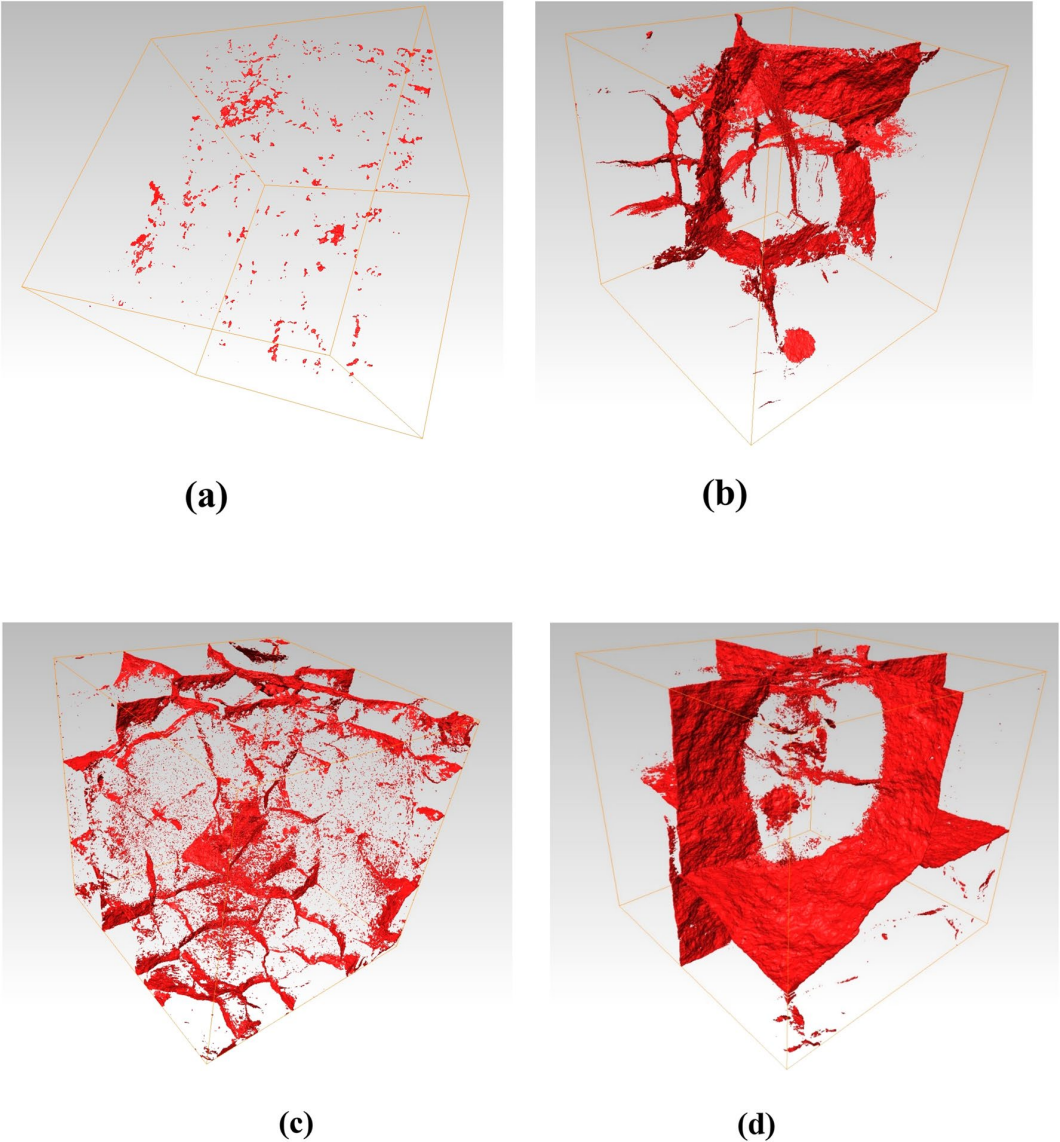
The spatial distribution of pores/cracks in the limestone sample after high-temperature treatment: (**a**) 700 °C; (**b**) 800 °C; (**c**) 900 °C; (**d**) 1000 °C.

**Figure 8:**
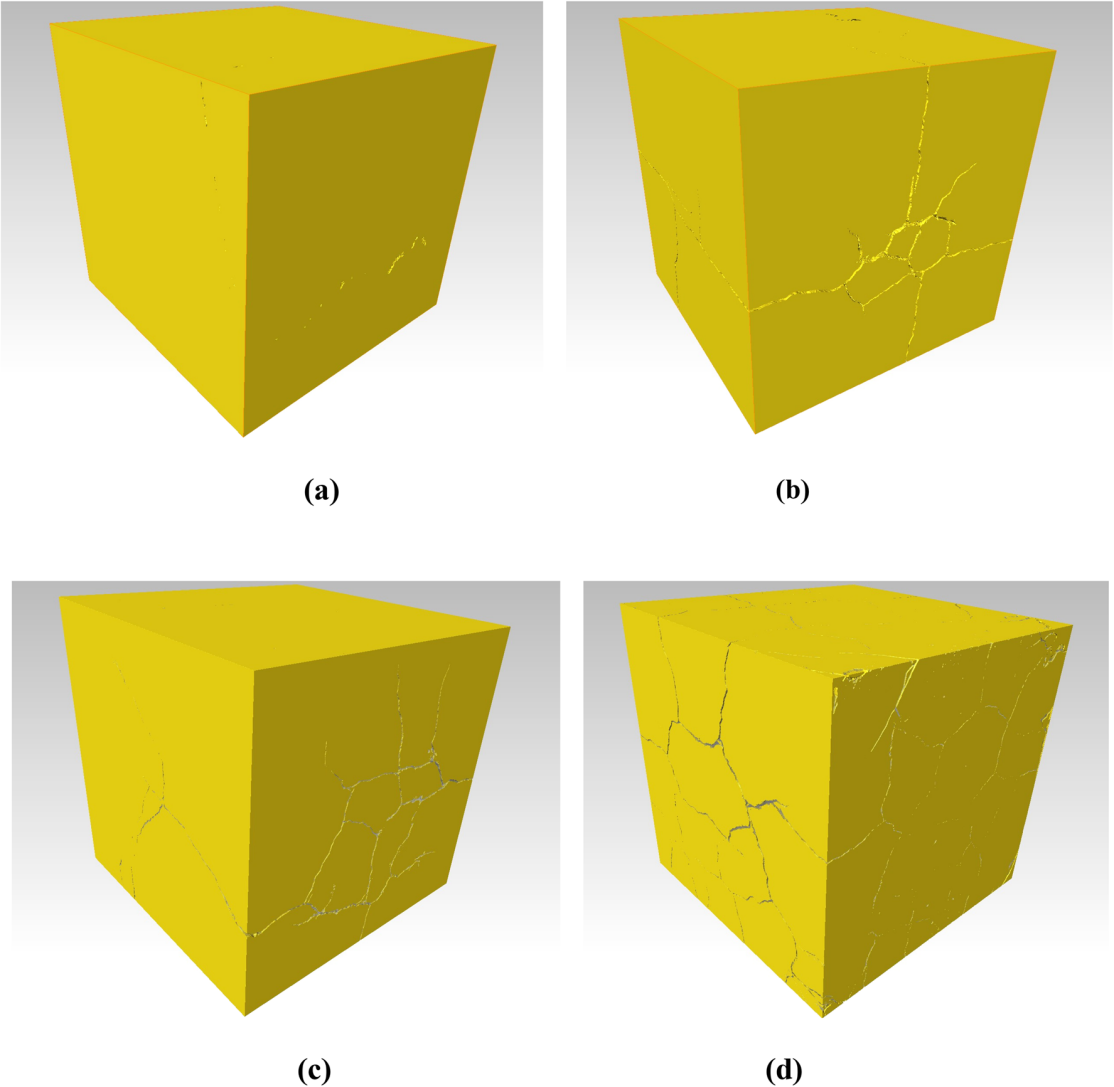
The spatial distribution of bones in the limestone samples after high-temperature treatment: (**a**) 700 °C; (**b**) 800 °C; (**c**) 900 °C; (**d**) 1000 °C.

Figures 7 and 8 show the three-dimensional spatial distribution of the pores and bones of the limestone samples treated at 700–1000 °C, respectively. A qualitative test conclusion can be obtained intuitively from the image: high-temperature has a significant effect on the internal pores of the limestone sample, and, exceeding a certain temperature threshold (for the Taihang Mountain limestone in this study, the temperature threshold is about 700 °C), internal pores rapidly develop, expand, and connect, reflecting a typical progressive destruction process from the inside to the outside.

### Analysis of porosity

**Figure 9:**
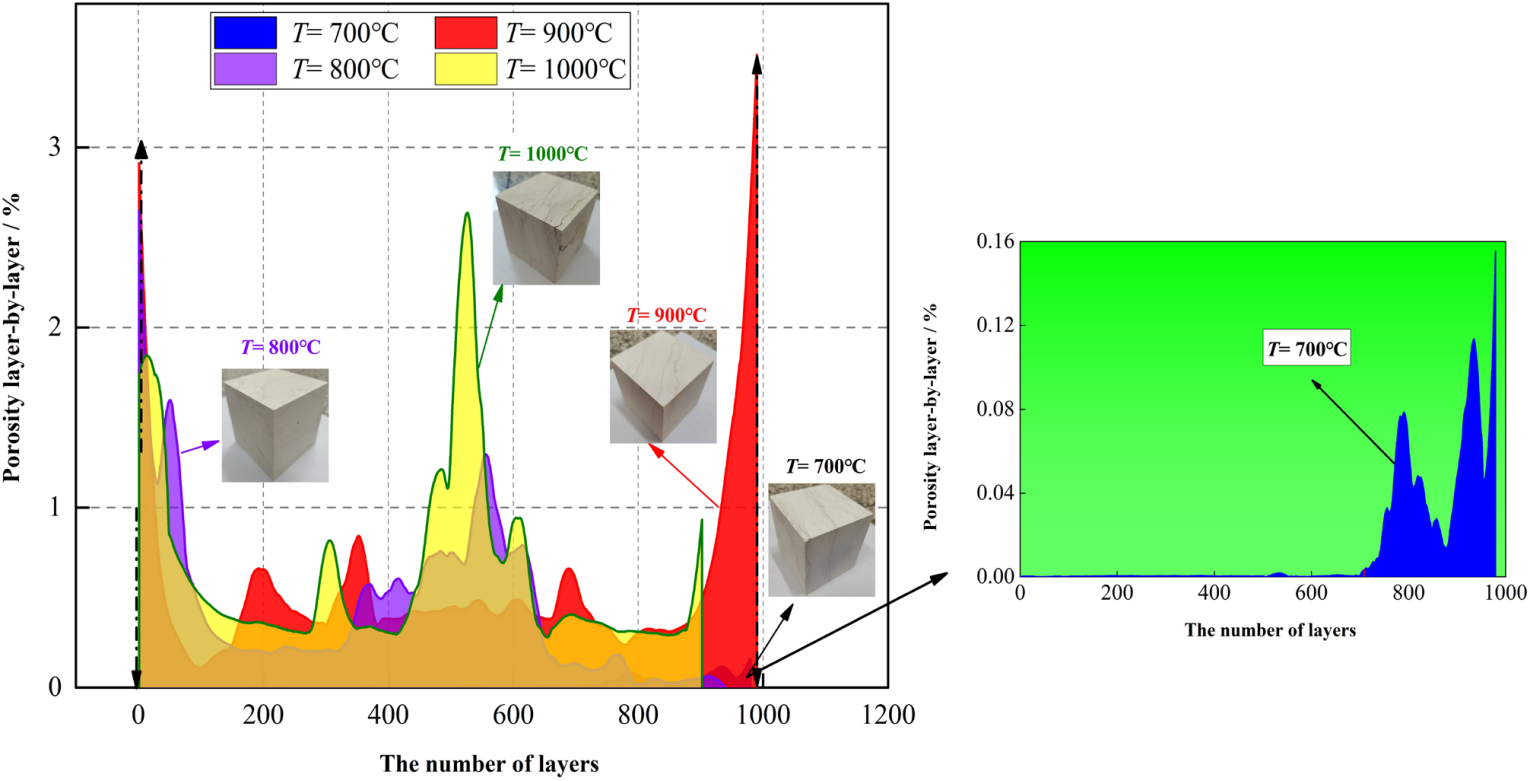
The layer-by-layer porosity distribution of limestone samples after high-temperature treatment.

**Figure 10:**
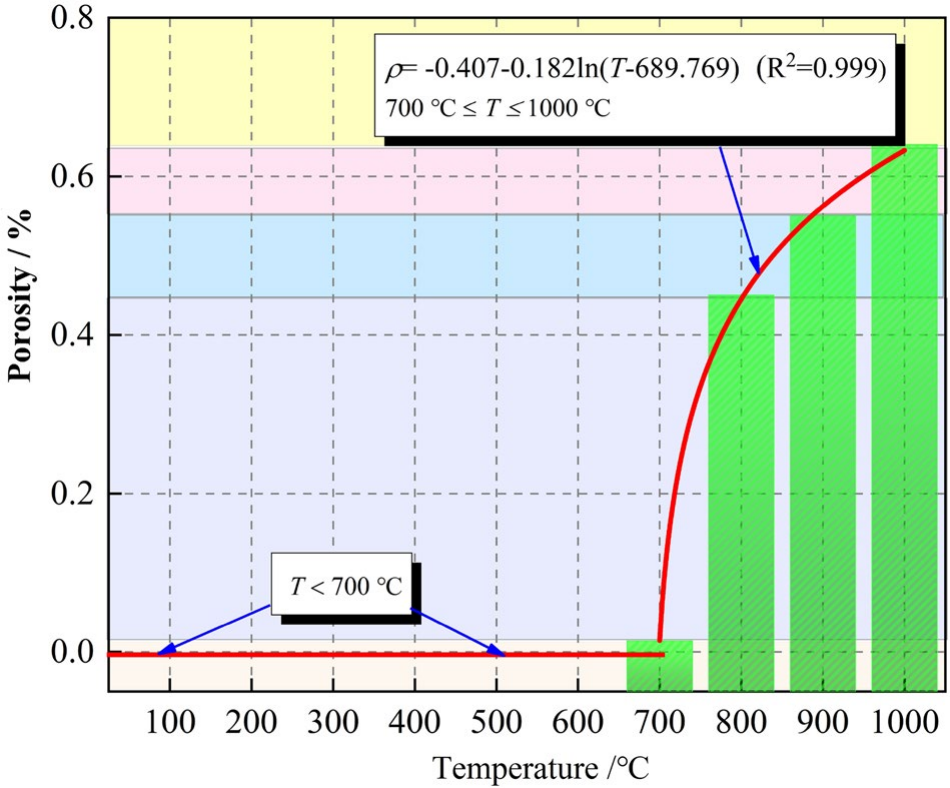
Variation of the porosity of limestone samples with temperature.

The layer-by-layer porosity means that the number of pixels occupied by the pores on each section in the Z direction calculated by the software, and the number of pixels occupied by the entire sample on each section in the Z direction can also be calculated. The ratio of the two is the porosity of the surface; thus, the change in porosity characteristics can be observed by the layer-by-layer porosity. The layer-by-layer porosity distribution of limestone samples after high-temperature treatment is shown in Fig. 9. The layer-by-layer porosity in the Z-axis direction fluctuates greatly, especially at the two ends of the sample near the edge. The volume porosity of the limestone sample after 700–1000 °C treatment is approximately 0.014%, 0.45%, 0.55%, and 0.64%, respectively. Based on the CT scan results, the fitting relationship between the volume porosity and temperature is constructed as:

1 $$ \rho  =  - 0.407 - 0.182\ln \left( {T - 689.769} \right)\quad \left( {700\,^{ \circ } {\text{C}} \le T \le 1000\,^{ \circ } {\text{C}}} \right) $$

where $\rho$ is the volume porosity of the limestone sample after high-temperature treatment, and *T* is the heating temperature. Figure 10 shows the change of temperature with volume porosity.

### Analysis of pore characteristics

**Figure 11:**
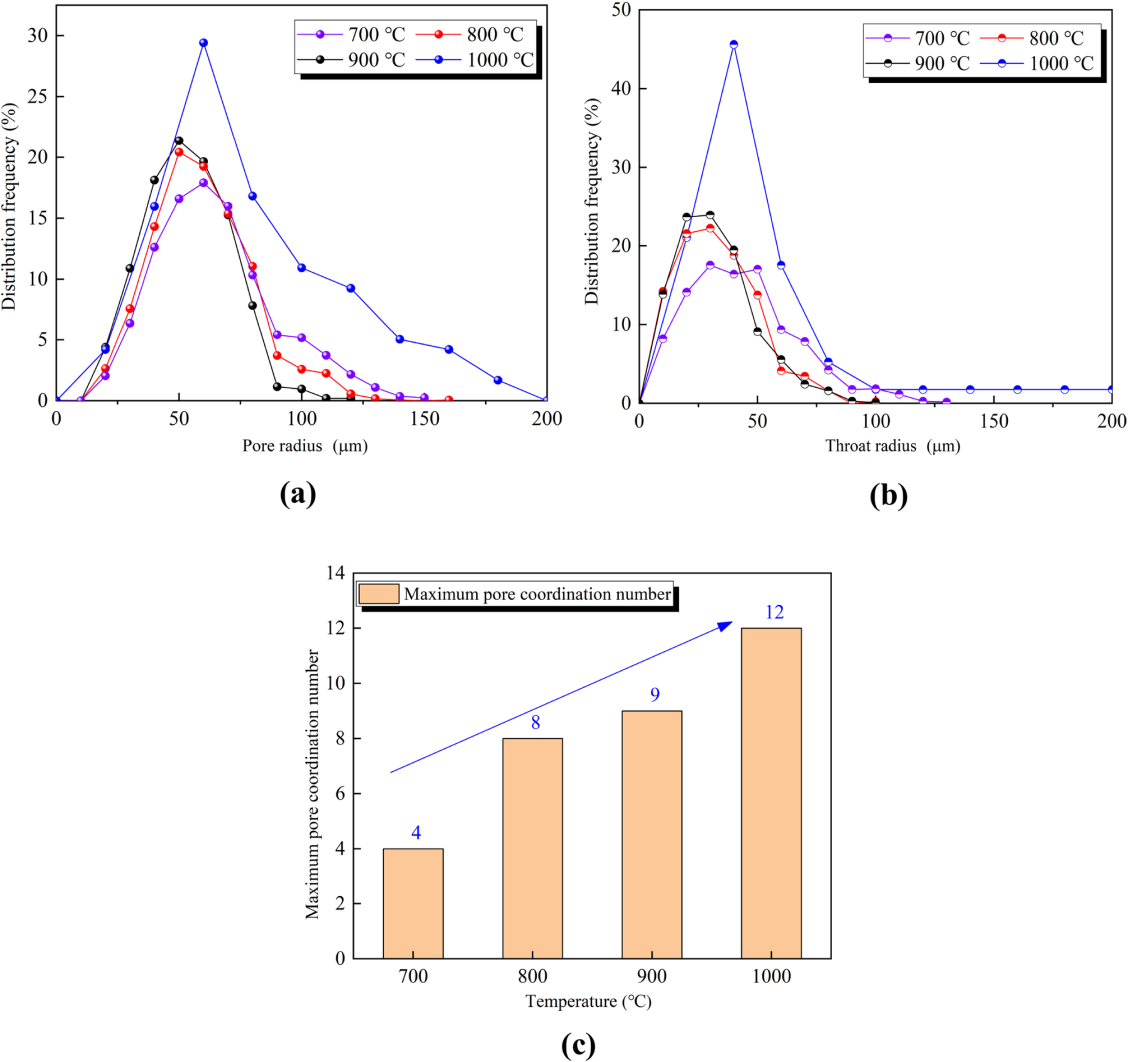
Frequency of pore characteristics distributions of limestone samples after high-temperature treatment: (**a**) Distribution of pore radius; (**b**) Distribution of throat radius; (**c**) Distribution of maximum pore coordination number.

The maximum sphere algorithm^38^ is used to extract pore and throat parameters from the reconstructed three-dimensional digital core image. The basic principle is to use any point in the pore pixel as the center of the sphere to continuously increase the radius and expand outward until it touches the rock skeleton, and all the pixels in the contained area are the largest sphere. The pore radius, throat radius and maximum coordination number were respectively used to characterize the characteristics of pores, throat and pore connectivity, and the frequency distributions of the pore structure parameters of limestone samples at different temperatures (700–1000 °C) were plotted, as shown in Fig. 11. It can be seen that high temperature has a certain influence on the microscopic characteristic parameters of the sample. With the increase of temperature, the pore radius, throat radius and maximum coordination number all show an increasing trend: At 700 °C, the pore and throat are skewed distribution, with relatively few large pores and throats. The pore radius is mainly distributed in the range of 20–60 μm, and the throat radius is mainly distributed in the range of 5-35 μm. The maximum coordination number is 4, and the throat and pore coordination are poor. With the increase of temperature to 800–900 °C, the distribution range and shape of pore radius and throat radius did not change significantly, but the proportion of large pores and throats gradually increased, and the maximum coordination number reached 8 and 9, respectively. When the temperature is 1000 °C, the pore radius distribution range extends to 30–80 μm, the throat radius extends to 5–50 μm, and the maximum coordination number further increases to 12. The maximum coordination number is the number of throats connected to a pore. The larger the maximum coordination number, the more the number of paired throats and the better the connectivity. Therefore, the increase in temperature improves the connectivity of the rock sample to a certain extent.

## Uniaxial mechanical test

**Figure 12:**
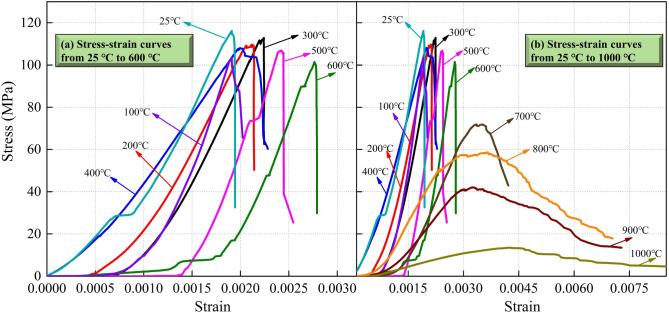
Stress–strain curves of limestone samples after high-temperature treatment: (**a**) 25–600 °C; (**b**) 25–1000 °C.

**Figure 13:**
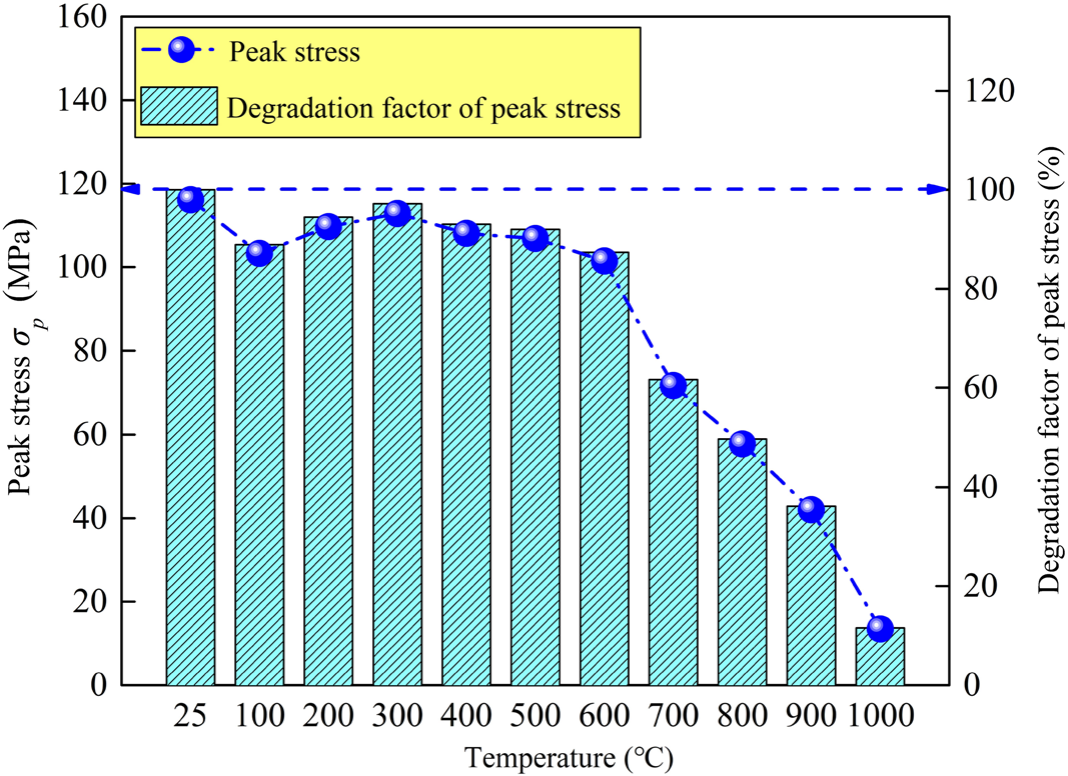
Variation of the peak stress and peak stress degradation factor of limestone samples with temperature.

A rock material testing machine is used to perform uniaxial mechanical loading test on the limestone samples after high-temperature treatment. The test adopts the displacement control mode and the loading rate is 0.0005 mm/s. The typical stress–strain curves are obtained, as shown in Fig. 12. It is found that the stress–strain curves of limestone after different high-temperature treatments show different characteristics, among which temperature plays a very important role: (1) When the temperature treatment range is 25–600 °C, samples exhibit brittleness characteristics; in other words, as the applied load increases, the sample falls rapidly after reaching the peak stress, the bearing capacity loses quickly, the failure is sudden, and the peak strain is relatively small. (2) When the temperature exceeds 700 °C, the samples exhibit plastic characteristics. The samples do not completely lose bearing capacity after reaching the peak stress. With the increase of the applied load, the sample still has a certain strength, and the failure is procedural, and there is a certain yield platform in the stress–strain curve. (3) After 700 °C treatment, the stress–strain curve shifts rapidly to the right, reflecting the increasing failure strain; the stress–strain curve drops rapidly, reflecting the decreasing failure strength, which also indicates that the damage effect of high temperature on rock is becoming more and more significant. The relationship between the peak stress and peak stress degradation factor (the ratio of the rock strength after high-temperature treatment to the rock strength at 25 °C) and the temperatures are plotted as shown in Fig. 13. The nonlinear relationship between rock strength and temperature:

2 $$ \sigma _{p}  = 105.03 + 8.05(T/100) - 1.71(T/100)^{2} \quad \left( {R^{2}  = 0.961} \right) $$

where $\sigma _{p}$ is the peak stress of the limestone sample after high-temperature treatment.

**Figure 14:**
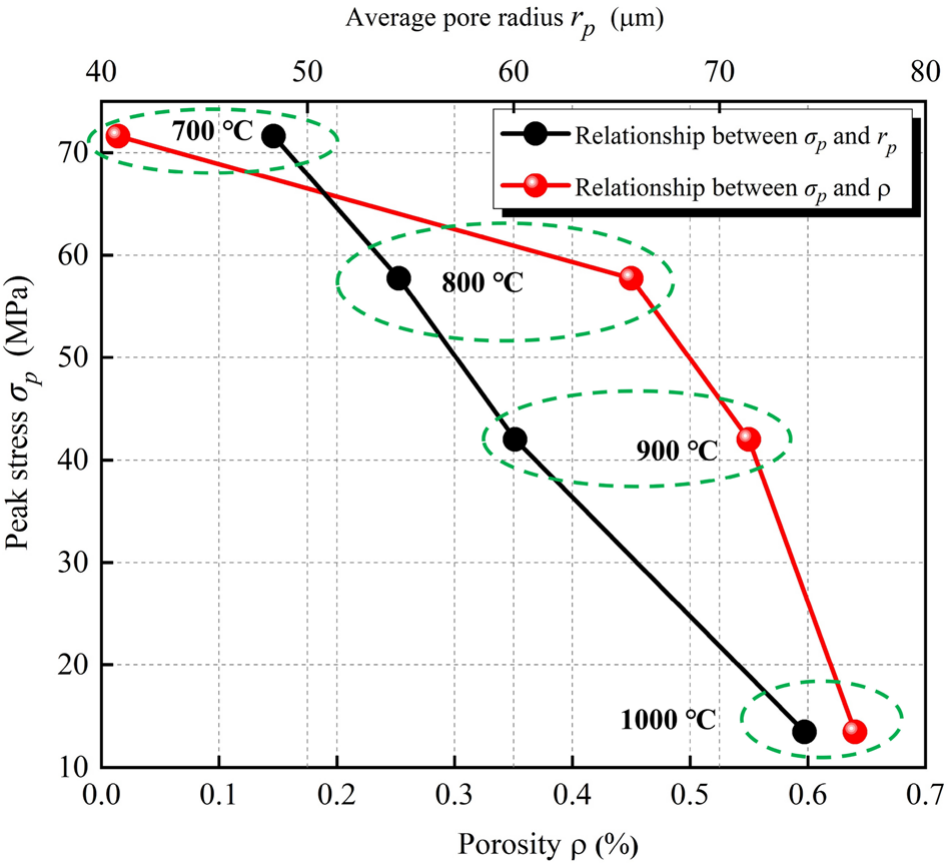
Relationship between porosity, average pore radius and peak stress of limestone samples after high-temperature treatment.

To analyze the relationship between strength and temperature, some typical laws can be explored: the rock strength at 25 °C is 116.11 MPa, which belongs to a hard rock; as the temperature increases, the strength decreases somewhat, but the fluctuation range is not large. When the treatment temperature reaches 600 °C, the strength still reaches 101.46 MPa, and the attenuation is 12.61% compared with normal temperature. However, when the treatment temperature reaches 700 °C, the strength rapidly decays to 71.61 MPa, and the attenuation is 38.33% compared with normal temperature. The conclusion is that the strength characteristics of limestone will undergo a sudden change around 700 °C. Draw the relationship between porosity, average pore radius and strength, as shown in Fig. 14. 700 °C is the mutation temperature of Taihang Mountain limestone selected in this study. This mutation temperature is not only reflected in the influence on the rock strength, when the mutation temperature is exceeded, the rock strength undergoes a sudden decay; it is also reflected in the brittle-plastic transition of rock, when the mutation temperature is exceeded, the rock changes from brittleness to plasticity. This macro-mechanical test, high-temperature test, and CT test are mutually confirmed to some extent. The representative indicators include: strength, stress–strain curve, apparent morphology and pore characteristics, which all have a similar trend with temperature.

## Discussions

There is no doubt that CT detection methods have obvious advantages in the quantitative spatial analysis of microscopic pores/cracks in rock samples. However, the existing problems are also prominent. The scale of microdefects in natural brittle materials like rocks is distributed at around 10^1^ μm. While studying meso-fracture from this scale is of practical engineering significance, the current resolution scale of industrial CT is about 10^2^ μm; therefore, a certain gap remains between the identification accuracy and the actual scale, which also causes errors between the indoor test results and the actual situations. Even if the accuracy of CT testing equipment can meet the requirements, it is difficult for rock samples to be processed into millimeter size due to its brittleness and discreteness. Moreover, contrasting to metal materials, whether rock samples of a small size can represent actual rock or even rock mass remains a controversy. The limestone sample used in this study was a relatively dense cube of 70 mm × 70 mm × 70 mm. In terms of indoor testing, this is a larger sample, and the test results of large-size samples are relatively more representative. However, the problem is that the accuracy of CT testing is hard to improve, the average precision is concentrated at 35.79 μm, and the result is that some smaller-scale microdefects cannot be identified. In conclusion, the accuracy of test equipment and the size of rock samples have become two major obstacles in the field of CT scanning. Therefore, much work remains to accurately characterize the damage of rock materials based on micro-fracture CT identification. The research in this study also reflects the above problems to some extent.

High temperature will cause damage to rock samples. The author has conducted some studies in this field^12,13,39^, including analysis of granite, sandstone, marble, etc., and found that the macroscopic mechanical behavior of rocks, especially the strength index, has a certain mutation phenomenon with the change of temperature. Based on the three-dimensional pore CT recognition study of limestone after high-temperature treatment in this research, the test results of sudden temperature change were also determined. For Taihang Mountain limestone, the mutation temperature of porosity was concentrated at about 700 °C or so, while the apparent morphology of the sample also changed significantly at about 700 °C. Furthermore, according to the mechanical test results, the samples will undergo an obvious brittle-plastic transition around 700 °C, and the strength will decay rapidly after exceeding this temperature. Therefore, although there are a series of subjective and objective factors, such as macro test error, sampling error, and CT test accuracy, the macroscopic and microscopic results displayed a consistent change trend in mutation temperature.

## Conclusions

From the results of the macro high-temperature heat treatment, the mutation temperature of limestone was approximately 700 °C. After the temperature exceeded the mutation temperature, the samples appeared milky white. As the temperature increased, the color gradually turned white, and macroscopic cracks began to appear on the surface. According to the three-dimensional reconstruction images of the meso-parameters, the high-temperature had a significant effect on the internal pores of the samples; when the temperature exceeded 700 °C, the internal pores of the samples rapidly developed, expanded, and connected, which reflected a typical progressive destruction process from the inside to the outside. The layer-by-layer porosity of the samples after high-temperature treatment fluctuated dramatically, especially at both ends of the sample near the edge. The volume porosity at 700–1000 °C was 0.014%, 0.45%, 0.55%, and 0.64%, respectively. The pore radius, throat radius and maximum coordination number all increase with the increase of temperature, indicating that high-temperature not only promotes the development of pores, but also improves the connectivity of pores. Based on the mechanical test results, it is found that when the temperature treatment range is 25–600 °C, the limestone exhibits typical brittle characteristics; when the temperature exceeds 700 °C, the limestone shows typical plastic characteristics, and there is a certain yield platform in the stress–strain curve. There are sufficient evidences to believe that 700 °C is the mutation temperature of the limestone selected in this study. This mutation temperature is not only reflected in its influence on the rock strength, but also reflected in the brittle-plastic transformation, when exceeding the mutation temperature, the rock strength suddenly decays, and the rock changes from brittleness to plasticity. This macro-mechanical test, high-temperature test and CT test are mutually confirmed to a certain extent.
